# Rehabilitation in the Intensive Care Unit for Adults with Traumatic Brain Injury: A Systematic Review and Meta-analysis

**DOI:** 10.1007/s12028-026-02551-x

**Published:** 2026-05-23

**Authors:** Fiona Howroyd, David McWilliams, Jonathan Weblin, Hon Sing Geoffrey Wu, Grace Turner, Fang Gao Smith, Tonny Veenith, Niharika A. Duggal, Zubair Ahmed

**Affiliations:** 1 Department of Inflammation and Ageing, School of Infection, Inflammation and Immunology, College of Medicine and Health, University of Birmingham, Birmingham, UK; 2 University Hospitals Birmingham NHS Foundation Trust, Birmingham, UK; 3 University Hospitals Coventry and Warwickshire NHS Trust, Coventry, UK; 4 School of Sport, Exercise and Rehabilitation Sciences, University of Birmingham, Birmingham, UK; 5 Royal Wolverhampton Hospital, Wolverhampton, UK; 6 Institute of Acute Care, University of Wolverhampton, Wolverhampton, UK; 7 Therapy Services, Queen Elizabeth Hospital Birmingham, Mindelsohn Way, Edgbaston, Birmingham, UK

**Keywords:** Head injury, Rehabilitation, Physical therapy, Mobilisation, Critical care

## Abstract

**Background:**

Traumatic brain injury (TBI) is a leading cause of disability worldwide. Rehabilitation in the intensive care unit (ICU) is a recommended intervention for the critically ill. However, the safety and effectiveness of physical rehabilitation in adults admitted to the ICU with TBI is currently unknown.

**Objectives:**

The primary objective was to evaluate the safety of physical ICU rehabilitation in adults with TBI, and secondary outcomes aimed to evaluate its effect upon physical outcomes and length of admission.

**Methods:**

Five electronic databases were searched up to 1 September, 2025, including studies investigating physical ICU rehabilitation interventions in adults with acute TBI. A narrative synthesis of safety outcomes was conducted owing to heterogeneity. Meta-analysis was performed for secondary outcomes using the random-effects model to compare pooled data for rehabilitation and no-rehabilitation groups. Risk of bias was assessed using the ROBINS-E tool for observational studies and the RoB2 tool for randomised studies.

**Results:**

Overall, 13 studies consisting of 3582 adults with TBI were included. Four studies reported safety outcomes, reporting no or low rates of adverse events. Meta-analysis identified an increase in the Perme ICU Mobility Scores at ICU discharge in those that received ICU rehabilitation [Mean Difference 3.25 (2.05–4.44), *p* < 0.00001]. Risk of bias assessment identified very high risk of bias in observational studies and some concerns of bias in randomised studies.

**Conclusions:**

Physical rehabilitation for adults with TBI in the ICU is potentially safe with suggested benefit upon mobility status at ICU discharge. Further high-quality studies of ICU rehabilitation for adults with TBI are required.

*Trial Registration*: PROSPERO CRD420251131380.

**Supplementary Information:**

The online version contains supplementary material available at https://doi.org/10.1007/s12028-026-02551-x.

## Introduction

Traumatic brain injuries (TBIs) are a major cause of death and disability across all ages, with a global annual incidence rate of 69 million [1]. TBIs are defined as brain tissue damage caused by external force to the head, which leads to a disruption of normal brain function [2]. TBI account for approximately 37% of all injury-related deaths across Europe, with the most prevalent mechanisms of injury including road traffic accidents and falls [3–5]. Survivors of TBI are affected by long-term physical disability, cognitive disorders and psychological disturbances, affecting functional independence and quality of life [6, 7]. As one of the most common causes of death and disability in adults aged under 45 years, TBIs have profound societal impacts and require urgent attention to improve short-term and long-term patient outcomes [6].

TBIs are highly complex and heterogenous injuries, with approximately half of all adults presenting to hospital with TBI requiring admission to the intensive care unit (ICU), regardless of TBI severity [8, 9]. In addition to the profound effects of TBI itself, patients with extended ICU admission and periods of immobility also face the extensive physical, cognitive and psychological consequences of critical illness [10, 11]. ICU-acquired muscle weakness (ICU-AW) is a generalised, symmetrical neuromuscular weakness that affects almost 50% of critically ill patients, leading to approximately 2% loss of skeletal muscle per day [12–14]. ICU-AW may result from critical illness polyneuropathy, myopathy, or a combination of both, and is characterised by diffuse muscle weakness, associated with systemic inflammation, bioenergetic failure and impaired muscle protein synthesis [12, 15]. ICU-AW is associated with prolonged ICU admission, increased ventilator days and poor long-term functional outcomes [10, 16–18]. With the average cost of an ICU bed in the UK National Health Service equating to more than £1500 per night, interventions to reduce the ICU care needs and sequalae of critical illness is of significant importance [19].

Rehabilitation can be defined as a set of interventions to optimise function and reduce disability [20]. Physical ICU rehabilitation may include multidisciplinary interventions such as passive and active exercise, early mobilisation and functional activities, aiming to prevent and overcome ICU-AW and long-term disability [21–23]. ICU rehabilitation is a complex intervention grounded in a multisystem pathophysiological framework, recognising that critical illness rapidly disrupts neuromuscular, cardiovascular, respiratory, metabolic and neurocognitive systems, and aims to overcome the biological sequalae of immobilisation and ICU-AW [24, 25]. Physical ICU rehabilitation modulates the development and severity of ICU-AW by preserving neuromuscular function and improving functional recovery [12, 26]. Although rehabilitation cannot fully counteract the catabolic and inflammatory mechanisms driving muscle loss in critical illness, it can preserve muscle mass, muscle strength and reduce the severity and functional consequences of ICU-AW [13, 24, 25].

When appropriately selected using structured safety thresholds and with appropriate physiological monitoring, physical rehabilitation in general ICU cohorts is safe and does not cause harm or undue risk; with a recent systematic review reporting a safety event incidence rate of 0.6% [27–29]. In general ICU populations, such as medical and surgical patients, rehabilitation delivered in the ICU is an effective intervention to prevent and overcome the physical and non-physical consequences of critical illness and is recommended in national ICU guidelines [30]. Previous studies in general cohorts have found numerous benefits of physical ICU rehabilitation, including reducing the period of mechanical ventilation and ICU admission, in addition to improving muscle strength and functional outcomes [31, 32]. In the long term, ICU rehabilitation improves strength and mobility and reduces healthcare resource utilisation and mortality [33, 34].

However, trauma and neurological injury guidelines often fail to mention rehabilitation in the ICU, whilst ICU guidelines fail to specify the needs of the TBI cohort [30, 35–39]. Furthermore, many landmark clinical trials of early ICU rehabilitation have excluded patients with TBI [40, 41]. The unique complexities of the neuro-trauma patient, including features such as altered conscious state, cognitive impairment and haemodynamic instability, can make ICU rehabilitation challenging in this patient group [42, 43]. Despite evidence supporting the idea that early rehabilitation is safe and beneficial for general ICU populations, there is limited understanding and consensus of the effectiveness of ICU rehabilitation specifically for TBI cohorts. This systematic review therefore aims to synthesise and evaluate the currently available literature for physical rehabilitation in the ICU for adult patients with acute TBI. The primary outcome of this systematic review was safety of physical ICU rehabilitation for adults with acute TBI, based upon reporting of adverse events, serious adverse events and adverse reactions. Secondary outcomes included physical outcomes and the length of ICU and hospital admission.

## Methods

This review has been reported following Preferred Reporting Items for Systematic Reviews and Meta-Analyses (PRISMA) guidelines (Supplementary File 1). The protocol for this systematic review has been registered with the international prospective register of systematic reviews (PROSPERO; CRD420251131380). The PROSPERO registration was completed during the early planning and pilot stages of this systematic review, and thus deviations from the protocol have been necessitated. Deviations from the protocol include: (1) refined primary and secondary outcomes; (2) inclusion of adults aged 16 years and over; (3) risk of bias assessment using the ROB2 tool for randomised studies, in addition to the ROBINS-E tool for observational studies; and (4) meta-analysis of outcomes reported by two or more studies, using mean difference (MD), when studies measure the same outcome, and standardised mean difference (SMD), when studies measure the same construct using different outcomes.

### Search Strategy

Five electronic databases including PubMed, Ovid Medline, AMED, CINAHL and EMBASE have been used to perform the systematic review for published studies from all years. The Population, Intervention, Comparator and Outcome (PICO) syntax framework was utilised to formulate and refine the search strategy, including adults hospitalised after acute TBI (all severities of TBI by any definition) and admitted to the ICU (population), receiving any physical rehabilitation intervention in the ICU (intervention), compared with those who do not receive ICU rehabilitation (comparator), investigating any type of outcome measure (outcome), including safety effects, physical outcomes and length of stay. A control group was not necessary for inclusion as the systematic review included both randomised and observational studies to ensure comprehensive synthesis of safety data. Searches were conducted using a combination of various keywords, synonyms, headings (MeSH) and entry terms for common search strings including “traumatic brain injury” AND “rehabilitation” AND “intensive care unit” within the title and abstract (Supplementary File 2). All searches were conducted by one author (F.H.) and checked by a second author (Z.A.).

### Study Screening

Duplicate records were removed prior to screening. Initial screening of the title and abstract of each study were completed using the software Rayyan by two independent reviewers (F.H. and Z.A.). Shortlisted studies were reviewed in full text and assessed for eligibility against a pre-defined inclusion and exclusion criteria. Since there were no disagreements, discussion between the two authors (F.H. and Z.A.) was not required to reach consensus.

### Eligibility Criteria

Only studies investigating physical rehabilitation interventions in the ICU for adult patients with TBI were considered. There was no limit to the location or year of publication. Only full-text, original articles published in the English language and in a peer-reviewed journal were considered; case reports, study protocols, conference abstracts, review articles and grey literature were excluded. The eligibility criteria included: (1) human studies; (2) adults (aged ≥ 16 years); (3) admitted to the ICU after acute TBI; (4) investigating any type of physical rehabilitation intervention delivered in the ICU (including any form of passive or active exercise or mobility programmes); and (5) any outcome measure, including safety events, physical outcomes and length of stay data. Rehabilitation interventions for cognition, sensory stimulation, speech and swallow were excluded, including music therapy, psychology and speech and language therapy, unless combined with physical rehabilitation activities.

### Data Extraction

Data extraction was completed by one reviewer (F.H.) and checked for accuracy by a second reviewer (Z.A.). Data collection of the key characteristics of each included study was performed using Microsoft Excel. Data extraction included author name, year of publication, country where the study was conducted, the rehabilitation intervention investigated and outcomes. Details of the rehabilitation intervention were collated on the basis of the Template for Intervention Description and Replication Checklist (TIDeR), including format, frequency, dose, timing of initiation, safety considerations and staff members responsible for rehabilitation [44]. Details of the population included age, TBI severity and gender. Where reported, the mean and standard deviation (SD) for each outcome measure and sample size for rehabilitation and no-rehabilitation groups were recorded for meta-analysis. If data was presented as median and interquartile range (IQR), data was converted to mean and SD (SD = IQR/1.35) [45].

### Risk of Bias Assessment

To assess the risk of bias, the ROBINS-E tool was used for observational studies and the RoB2 tool for randomised studies [46, 47]. The risk of bias was assessed by two authors independently (F.H. and Z.A.). For the ROBINS-E tool, each study was assessed from low to very-high-risk against seven criteria, with a judgement of overall risk of bias based on the highest rating for each study. The RoB2 tool followed an algorithm for overall risk of bias, based upon the judgement of risk against five categories from low to high. There were no discrepancies between the authors that required further discussion to resolve.

### Analysis and Synthesis

Meta-analysis was performed using the Review Manager software (version 5.4, Cochrane Informatics & Technology, London, UK). Pooled data from the included studies was analysed using the continuous data function and inverse variance weighting within a random-effects model to compare rehabilitation and no-rehabilitation groups, when reported in two or more studies. The MD was selected when studies measured the same outcome and SMD was selected when studies measured the same construct using different outcomes. The MD or SMD, 95% confidence intervals (CI) and *p*-values for the test of the overall effect, along with *I*^2^ statistics were reported for each outcome. Descriptive data of patient demographics were reported as mean and standard deviation (SD). A *p*-value ≤ 0.05 was deemed as statistically significant throughout.

Narrative synthesis was performed where quantitative data synthesis and meta-analysis was not possible because of low study numbers or methodological heterogeneity, owing to, for example, variance in study design and outcome measures [48]. A preliminary synthesis used tables and a short textual description of each study using the TIDeR checklist to develop common themes [44].

## Results

**Fig. 1:**
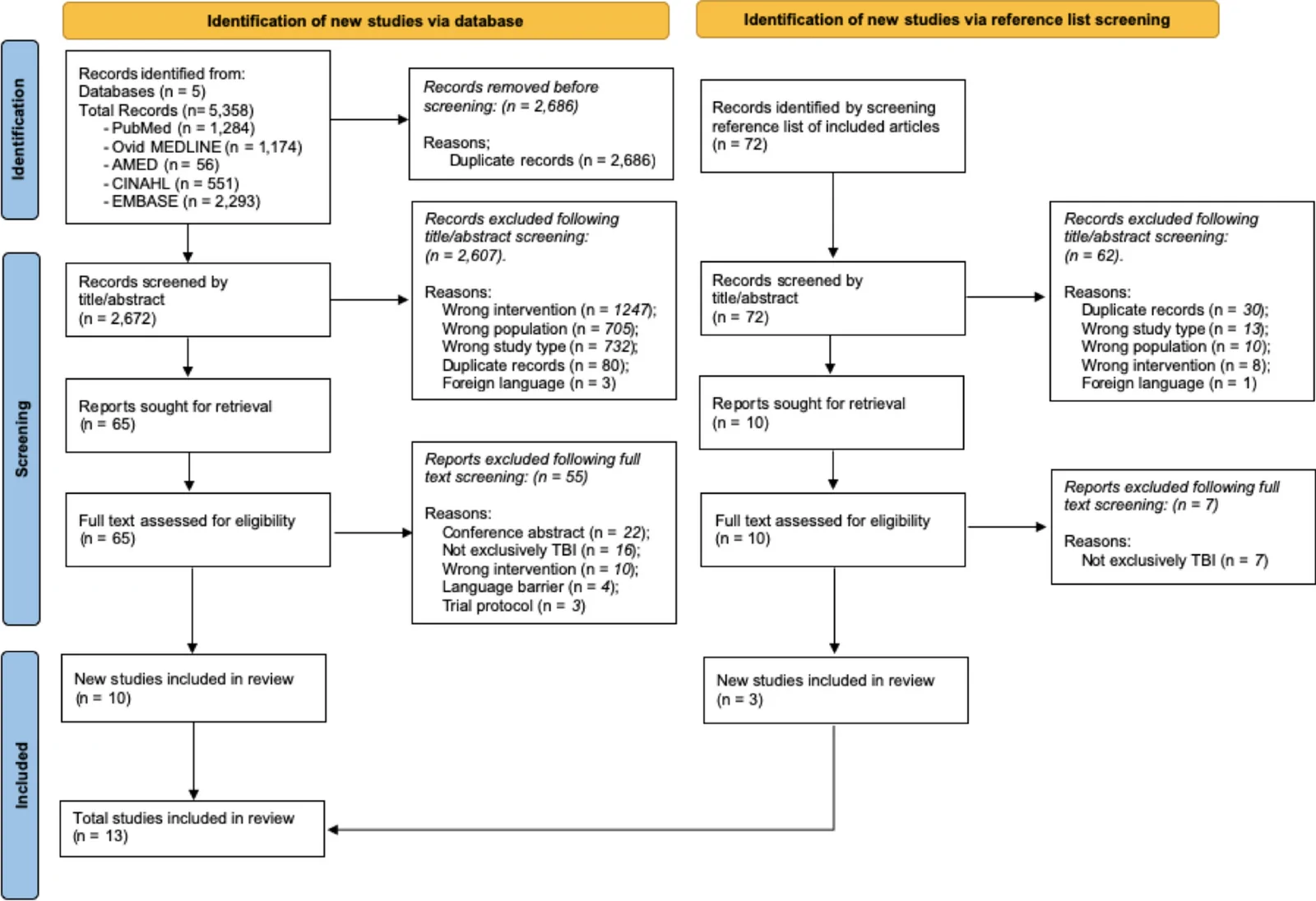
PRISMA flowchart of study identification, screening and inclusion

The study selection and screening process is presented in Fig. 1. The literature search retrieved 5358 articles from five electronic databases. After duplicates were removed and preliminary screening of titles and abstracts, 65 studies remained for full text review, of which ten studies were eligible for inclusion. Reference-list screening of the included studies identified three further studies. The key characteristics of the final 13 included studies are summarised in Table 1. The review included 3582 adult patients with TBI, of which 69.8% (*n* = 2500) were male with a mean age of 48.02 ± 7.72 years in those studies that reported age and sex.

**Table 1:** Details of included studies

First author Surname, year (reference)	Location	Study design	Population			Intervention	Control	Primary outcomes	Sample size (***n*)**
			TBI severity	Age	Males *n* (%)/females *n* (%)				
Alvsåker, 2023 [40]	Norway	Retrospective observational	Mod–severe (GCS 3–14)	47 ± 2.9	M = 759 (75.7%) F = 244 (24.3%)	Interdisciplinary mobility and ADL training	Standard of care	Discharge destination, ICU and hospital LOS	1003
Alvsåker, 2025 [41]	Norway	Retrospective observational	Mod–severe (GCS 3–14)	46 ± 19.3	M = 533 (76.4%) F = 165 (23.6%)	Interdisciplinary mobility and ADL training	Standard of care	Mortality, ICU and hospital LOS	698
Cetin, 2024 [42]	Turkey	Retrospective cohort study	Any TBI	54.7 ± 21.1	M = 39 (83%) F = 8 (17%)	Individualised PT activities	Pre- and post-intervention	GCS, GOSE, BI, DRS, MSAS, FAC	47
Elkbuli, 2022 [43]	USA	Retrospective observational	Any TBI	61.8 ± 3.3	M = 732 (64.5%) F = 403 (35.5%)	PT mobilisation with initiation < 24 h	Initiation at 24–48 h, 48–72 h or > 72 h	Discharge destination	1135*
Lima, 2022 [44]	Brazil	Prospective, single-blind, controlled trial	Any TBI	49.5 ± 13.5	M = 37 (%) F = (%)	Mobilisation protocol based on arousal level	Acquired brain injury	GCS and IMS	58
Lippert-Grüner, 2010 [45]	Germany	Prospective observational	Severe TBI (GCS < 8)	39 ± 43.7	M = 21 (77.8%) F = 6 (22.2%)	Multimodal sensorimotor therapy	Nil	GOS, BI, FIM	27
Mehta, 2017 [46]	India	Observational cross-sectional	Any TBI	35 ± 25.9	M = 27 (90%) F = 3 (10%)	Body positioning	Pre- and post-positioning	Vital parameters	30
Nagarathinam, 2019 [47]	India	Interventional cohort	Any TBI	NR	NR	Passive transfer to chair	30 versus 60 min of sitting in chair	CRS-R	48
Riberholt, 2021 [48]	Denmark	Randomized feasibility	Severe TBI (GCS < 11)	43 ± 6	M = 27 (71.1%) F = 11 (28.9%)	Tilt-table mobilisation	Standard care	Feasibility and safety events	38
Steiner, 2016 [49]	Austria	Historical observational	Severe TBI (GCS ≤ 8)	40.9 ± 4.7	NR	PT/OT/SLT	Standard rehabilitation	GCS, PTA duration, FIM, GOSE, CIQ	62
Wu, 2024 [50]	China	Historical control	Any TBI	51.7 ± 16.3	M = 220 (77.2%) F = 65 (22.8%)	Consciousness-guided early mobility	Retrospective controls	Diagnosis of pneumonia	285
Yen, 2022 [51]	Taiwan	Pre-post intervention	Mod–severe (GCS 6–13)	55.5 ± 0.3	M = 62 (72.1%) F = 24 (27.9%)	Progressive mobility protocol	Retrospective controls	Perme ICU Mobility Score and rate	86
Yen, 2024 [52]	Taiwan	RCT	Mod–severe (GCS 6–13)	52.1 ± 1.3	M = 43 (66.2%) F = 22 (33.8%)	Out-of-bed mobility	Upright positioning in bed	Perme ICU Mobility Score, FIM and LOS	65

ADL, activities of daily living; BI, Barthel Index; CIQ, Community Integration Questionnaire; CRS-R, Coma Recovery Scale; DRS, Disability Rating Scale; F, females; FAC, Functional Ambulation Classification; FIM, Functional Independence Measure; GCS, Glasgow Coma Scale; GOS, Glasgow Outcome Scale; GOSE, Extended Glasgow Outcome Scale; h, hours; ICU, intensive care unit; IMS, Intensive Care Unit Mobility Scale; LOS, length of stay; M, males; MSAS, Mobility Scale for Acute Stroke; Mod, moderate; *n*, number; NR, not recorded; OT, occupational therapy; PT, physiotherapy; PTA, post-traumatic amnesia; RCT, randomised controlled trial; SLT, speech and language therapy. *Sample size of participants included in the study with TBI

Reporting of a TBI diagnosis was inconsistent across studies, yet common characteristics included clinical history, admission Glasgow Coma Scale (GCS) scores and imaging. Severity of TBI varied between studies, with six studies including all severities of TBI, four including moderate–severe and three including severe TBI only; however, classification of severity varied between studies (Table 1). Seven of the included studies recorded admission GCS (weighted mean GCS 7.89 ± 5.76, *n* = 2178). Five studies recorded admission GCS in both the rehabilitation and control groups, demonstrating a statistically significant difference in the weighted mean GCS scores between groups (7.28 ± 5.15, *n* = 1151 vs. 8.55 ± 6.39, *n* = 818; *p* < 0.0001), rehabilitation vs. control, respectively.

### Risk of Bias

**Fig. 2:**
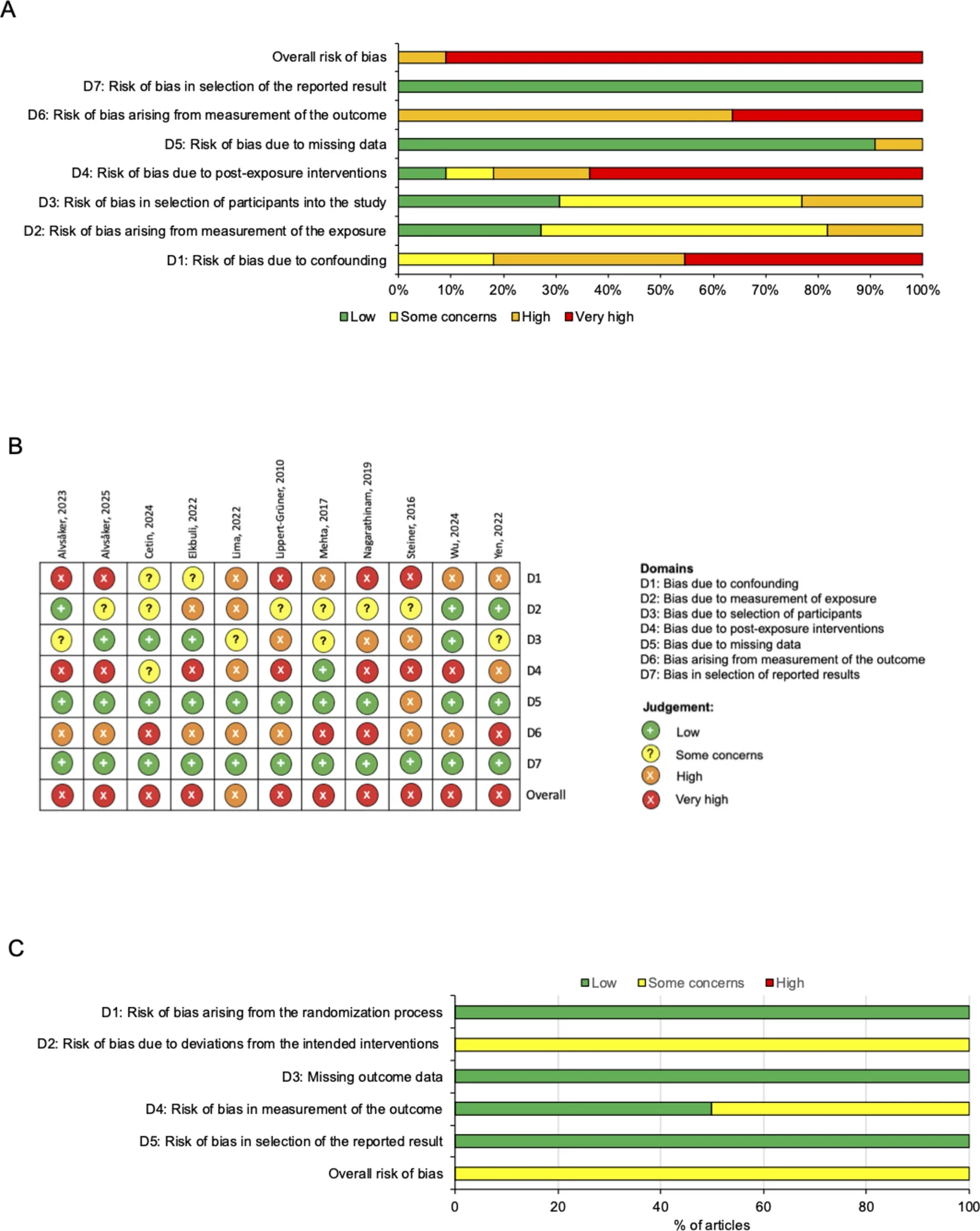
**A** Risk of bias summary chart assessed using the ROBINS E-tool for observational studies (*n* = 11) in the all studies chart. **B** Risk of bias assessed using the ROBINS E-tool for observational studies (*n* = 11) in individual studies against the seven criteria. **C** Risk of bias summary chart assessed using the RoB2 tool for randomised studies (*n* = 2)

Risk of bias analysis demonstrated an overall judgement of high to very-high risk of bias in observational studies (*n* = 11) (Fig. 2A and B) and some concerns in randomised studies (*n* = 2) (Fig. 2C). All of the observational studies (100%) were judged as having high to very-high risk of bias (Fig. 2A), with all (100%) of the studies judged as high to very-high risk due to bias in measurement of the outcome, over half of all studies judged as very-high risk due to bias in post-exposure interventions (63.6%), over a third due to confounding (45.5%) and measurement of the outcome (36.4%) (Fig. 2A and B). Although three of the five categories were deemed as low risk in the assessment of randomised studies, both studies had some concerns of bias owing to deviation from the intended intervention (Fig. 2C).

### Safety of ICU Rehabilitation for Adults with Acute TBI

Four studies reported on safety outcomes [49–52]. Two studies reported no serious adverse events or adverse events associated with ICU mobilisation interventions [49, 50]. A randomised feasibility trial of an early tilt-table stepping device intervention reported 24 serious adverse events, 73 adverse events and 4 adverse reactions in 17 of the 19 patients included in the intervention group, yet with no significant differences in the number of events between groups (a total of 101 events in the intervention group and 101 events in the control group) [51]. Examples of the recorded serious adverse events included pneumonia, delirium, sepsis and seizure, and adverse events included nasal gastric tube removal, pressure ulcer, urinary tract infection and vomiting. A historic controls study of a mobilisation strategy reported 19 unexpected events, including falls or the infusion needle falling out, and 59 stop events, such as a change in vital signs or dizziness, during 2863 rehabilitation interventions, involving 36 of the 86 patients in the intervention group [52]. Overall, physical ICU rehabilitation was associated with a safety event rate of 5.8% (179 events across 3066 rehabilitation sessions). However, safety event rates are based upon the reporting of the number of rehabilitation sessions and safety events in the rehabilitation group of two study studies only, with inconsistent reporting methods between studies.

### The Effect of ICU Rehabilitation upon Physical Outcomes After Acute TBI in Adults

In total, 27 physical outcomes were identified and categorised into six themes: mobility status, functional status, muscle function, neurological disability, vital parameters and complication rates (Supplementary File 3). Owing to heterogeneity across outcome measures and low study numbers, a meta-analysis was only possible for the themes of mobility status, functional status, neurological disability and muscle function.

#### Mobility Status

**Fig. 3:**
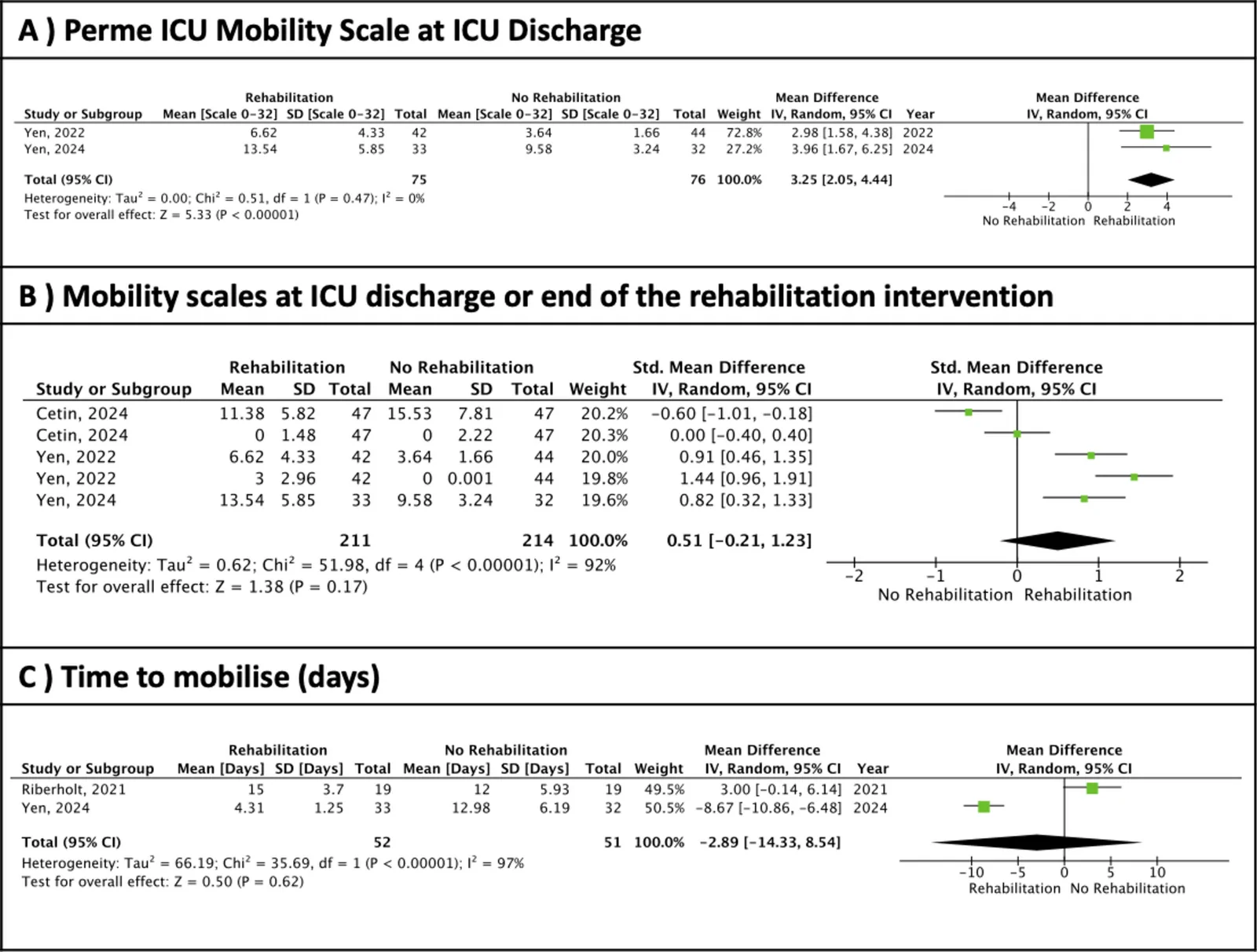
Meta-analysis of pooled data comparing rehabilitation with no-rehabilitation groups. **A** Perme ICU Mobility Scale at ICU discharge. **B** Mobility scales measured at the end of the rehabilitation intervention or at ICU discharge (Cetin 2024: Mobility Scale for Acute Stroke and Functional Ambulation Classification; Yen 2022: Perme ICU Mobility Scale and Modified Mobility Scale; Yen 2024: Perme ICU Mobility Scale). **C** Time to mobilise (days). ICU, intensive care unit

Adult patients with TBI who received rehabilitation in the ICU had significantly higher Perme ICU Mobility Scores at ICU discharge compared with those who did not receive ICU rehabilitation (MD 3.25, 2.05–4.44, *p* < 0.00001) (Fig. 3A). It is of importance to note that outcomes were reported in just two studies (*n* = 2) by the same authors, but in different populations over different time periods. When combining mobility status outcomes, there are suggestions from the data that patients with TBI who received ICU rehabilitation may have improved mobility status at the point of ICU discharge, but these changes were not statistically significant (SMD 0.82, 0.32–1.33, *p* = 0.17) (Fig. 3B). Results from two included randomised controlled trials (RCTs) also suggested that patients who received rehabilitation in the ICU, may have commenced mobilisation quicker than those in no-rehabilitation control groups, but again, results were not statistically significant (MD − 2.89 days, − 14.33 to − 8.54, *p* = 0.62) (Fig. 3C). In summary, despite a limited number of studies suggesting a possible improved Perme ICU Mobility Score, there is not enough high-quality evidence to support the notion that ICU rehabilitation improves mobility status in patients with TBI.

#### Functional Status, Muscle Function and Neurological Disability

**Fig. 4:**
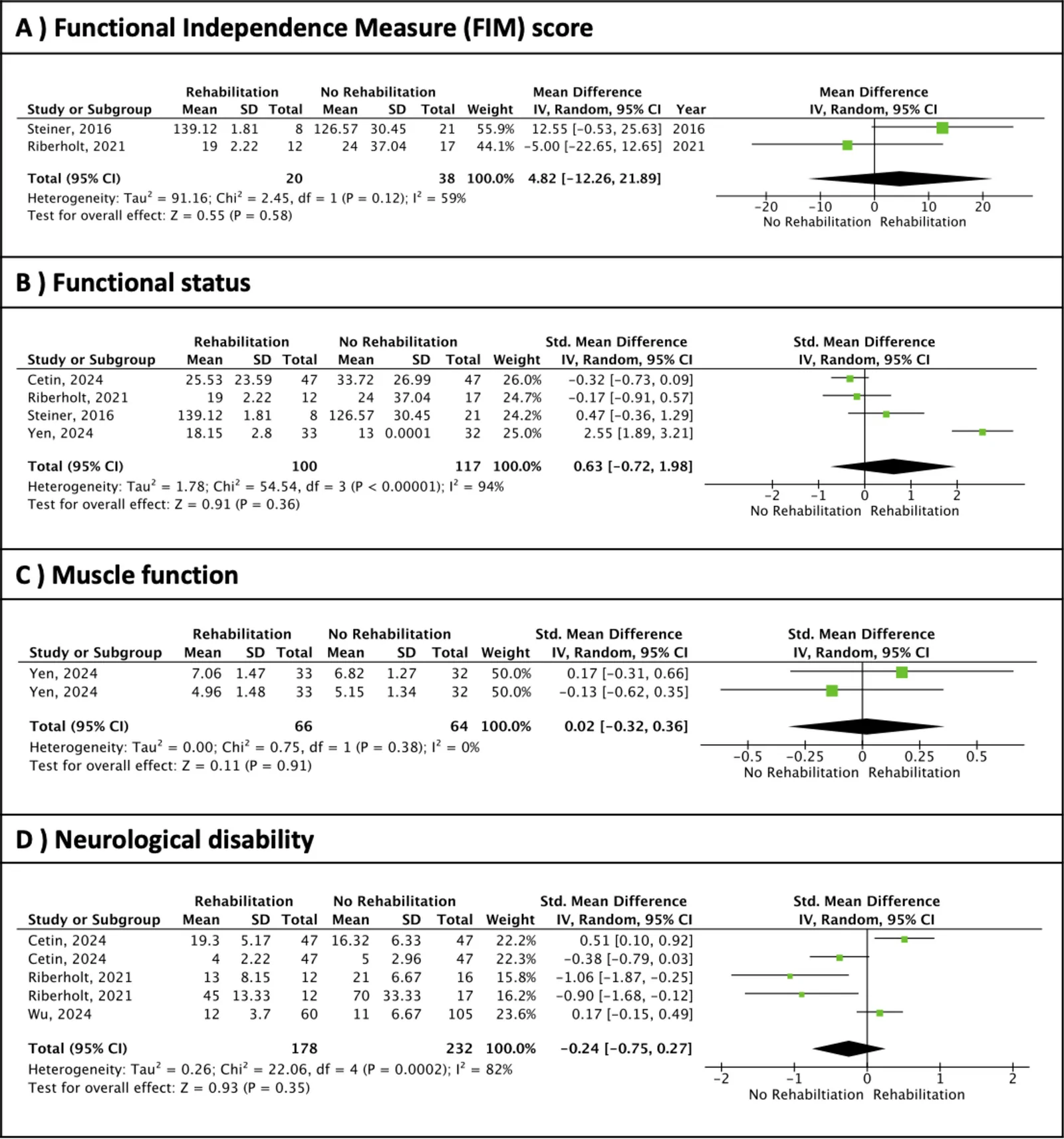
Meta-analysis of functional status, muscle function and neurological disability. Meta-analysis of pooled data comparing rehabilitation with no-rehabilitation groups at the end of the rehabilitation intervention or at ICU discharge. **A** Functional Independence Measure (FIM). **B** Functional status (Cetin 2024: Barthel Index; Riberholt 2021: FIM; Steiner 2016: FIM, Yen 2024: FIM Motor Score). **C** Muscle function (Yen 2024: Skeletal Muscle Index and Phase Angle). **D** Neurological disability (Cetin 2024: Glasgow Outcome Scale Extended and Disability Rating Scale; Riberholt 2021: Coma Recovery Scale and Early Functional Ability Score; Wu 2024: Glasgow Coma Scale)

**Fig. 5:**
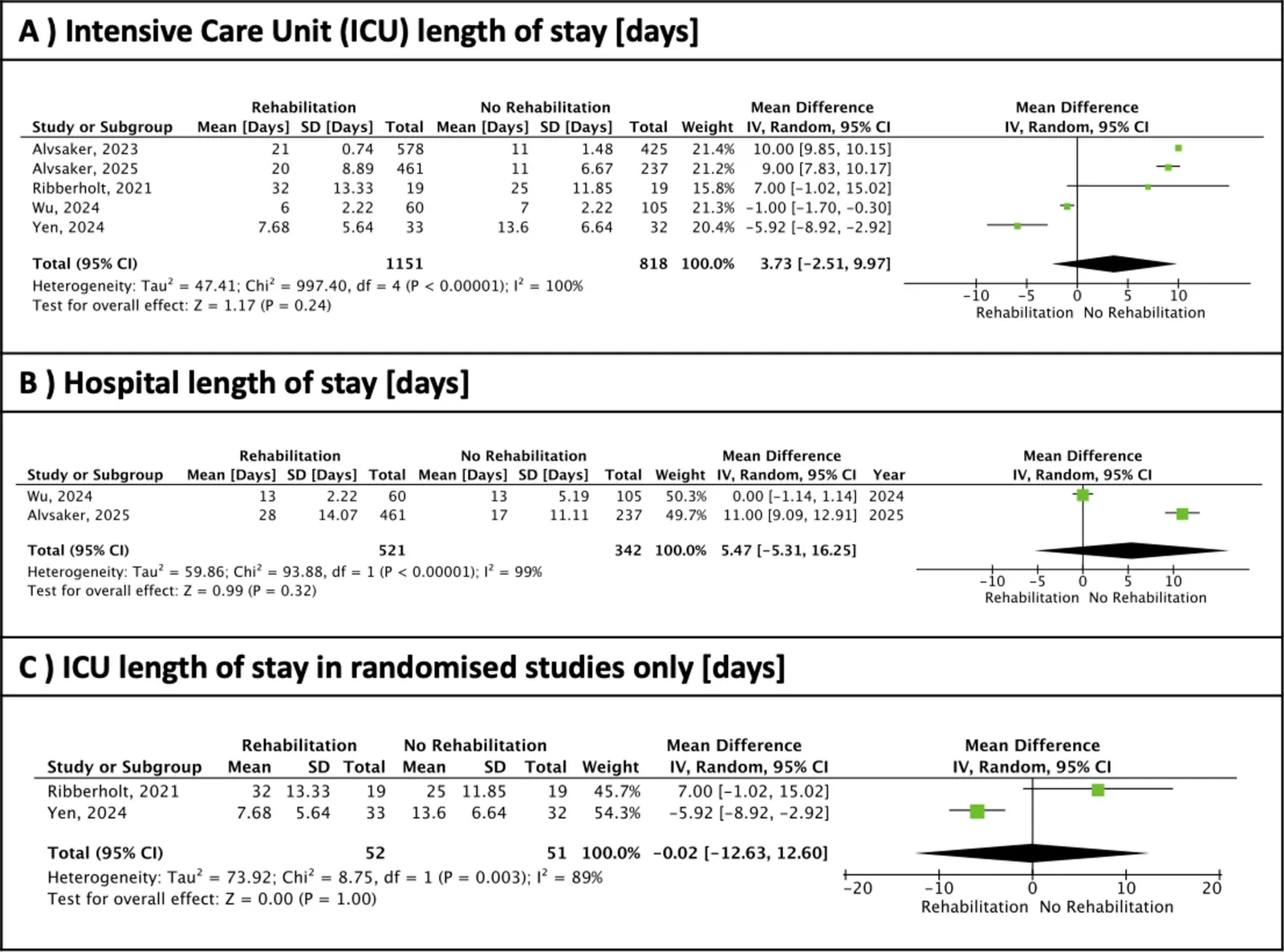
Meta-analysis of pooled data comparing length of stay data (days) in rehabilitation with no-rehabilitation groups. **A** Intensive care unit (ICU). **B** Hospital. **C** ICU in randomised studies only

Adult patients with TBI who received ICU rehabilitation showed a non-significant improvement in functional independence measure (FIM) scores (MD 4.82, − 12.26 to − 21.89, *p* = 0.58) (Fig. 4A), functional status (SMD 0.63, − 0.72 to − 1.98, *p* = 0.36) (Fig. 4B) and muscle function (SMD − 0.02, − 0.32 to − 0.36, *p* = 0.91) (Fig. 4C) at the point of ICU discharge compared with those who did not receive ICU rehabilitation. In addition, adult patients with TBI who received ICU rehabilitation had worse levels of neurological disability at ICU discharge, compared with those who did not receive ICU rehabilitation; however, results were also not statistically significant (SMD − 0.24, − 0.75 to − 0.27, *p* = 0.35) (Fig. 5D). In summary, despite results indicating improved FIM scores, functional status and muscle function, and worse neurological disability in patients with TBI who received ICU rehabilitation, results were not significantly different and reported in low study numbers (*n* = 2–5), making results unreliable.

### The Effect of ICU Rehabilitation upon Length of Admission

Trends suggest that adult patients with TBI who received ICU rehabilitation had a longer ICU length of stay (MD 3.73 days, − 2.51 to 9.97 days, *p* = 0.24) (Fig. 5A) and a longer hospital length of stay (MD 5.47 days, − 5.31 to 16.24 days, *p* = 0.32) (Fig. 5B) compared with those who did not receive ICU rehabilitation; however, the results were not statistically significant. Results remained statistically nonsignificant when measuring ICU length of stay in randomised studies only, although in favour of rehabilitation (MD − 0.02 days, − 126.3 to 12.60 days, *p* = 1.00) (Fig. 5C). It is of importance to note that each of the outcomes were reported in low study numbers (*n* = 2–5) with high levels of heterogeneity (*I*^*2*^ = 89–100%).

### ICU Rehabilitation Interventions for Adult Patients with TBI

Narrative synthesis using the TIDeR checklist framework identified a breadth of physical ICU rehabilitation interventions (Supplementary File 4) [44].

#### Format and Staff-Members

The most common intervention was out-of-bed mobilisation (*n* = 10 studies). However, mobilisation techniques varied from passively transferring patients from a bed to a chair or tilt-table device, to active mobility protocols in which patients aimed to progress to walking independently unaided. Other therapies included training in activities of daily living, positioning, passive and active limb and trunk exercises, balance training and family engagement. Rehabilitation was delivered by a variety of healthcare professionals, with the most common being physiotherapists (also termed physical therapists) (*n* = 12) and occupational therapists (*n* = 4). Four studies involved interdisciplinary and multi-component interventions, also including speech therapists, ICU nurses and physicians.

#### Frequency, Timing and Dose

The frequency of rehabilitation varied from 5 to 7 days per week, once to twice per day. The duration of each session varied from 15 to 60 min, yet two studies recommended 2–5 h of rehabilitation per day. Most studies (*n* = 11) used clinical parameters to determine the initiation of rehabilitation (such as after cessation of sedation or when haemodynamically stable), yet some studies also commenced rehabilitation at specific timepoints, varying from less than 24 h, up to 19.82 ± 17.9 days after admission. Seven studies suggested that the rehabilitation programme was individualised and tailored to the patient (e.g. dependent upon a patients’ response to exercise or neurological status).

#### Safety Considerations

Eleven studies detailed explicit inclusion and exclusion criteria. Inclusion was based upon haemodynamic stability in nine studies. Common exclusion criteria included spinal cord injury; spinal, pelvic or long-bone fractures; cardiac arrythmias; and uncontrolled seizures or epilepsy. Eight studies suggested patient monitoring of vital signs during rehabilitation interventions with five studies reporting explicit criteria for patient observations, including blood pressure, intracranial pressure, heart rate, mean arterial pressure, respiratory rate, saturations of oxygen and the levels of positive end expiratory pressure and fraction of inspired oxygen. Seven studies defined a specific stop criterion when rehabilitation should be ceased, including deterioration of vital observations, pain or adverse events, such as falls, unplanned extubation or invasive tube dislodgement.

## Discussion

The aim of this systematic review was to determine the safety and effectiveness of physical rehabilitation in the ICU for adult patients with TBI upon physical outcomes and length of ICU and hospital admission. Thirteen studies, consisting of 3582 patients with TBI, were included. A number of rehabilitation interventions were identified, yet predominantly comprised of out-of-bed mobilisation delivered by physiotherapists. Results suggest that physical ICU rehabilitation may be safe for adult patients with TBI, however, safety events were only reported in four of the included studies. Meta-analysis identified that physical ICU rehabilitation may be associated with increased Perme ICU Mobility Scores at ICU discharge. Trends suggest that physical ICU rehabilitation may also be associated with improved functional status, mobility status and muscle function, yet trends also suggest an increase in ICU and hospital length of stay and worse neurological disability; however, results were not statistically significant. Overall, the low study numbers, including the low number of randomised-controlled trials, and the high risk of bias affects the validity of our findings. Further high-quality, prospective studies of physical rehabilitation in the ICU for adults with TBI are therefore urgently required in order to benefit policy and clinical practice.

The primary aim of this systematic review was to establish the safety of physical ICU rehabilitation for adults with acute TBI. Results suggest that physical ICU rehabilitation may be a safe intervention for adult patients with TBI, yet safety reporting was overall limited across the included studies. Four of the included studies reported safety events during rehabilitation, including three studies of progressive mobility programmes and one utilising passive tilt-table transfers [49–52]. At baseline, up to 98% of patients in the rehabilitation groups of these studies were mechanically ventilated, up to 50% required an ICP bolt and 40% required neurosurgical management, suggesting an acute and critically ill population of neurotrauma patients. However, patient characteristics were not recorded at the time of mobilisation, and thus, interpretation and clinical application of safe rehabilitation practice after TBI is limited. Nonetheless, rehabilitation was deemed safe and feasible amongst a small cohort of acute TBI patients in ICU, with out-of-bed mobilisation commencing between 4 and 15 days after ICU admission, once patients had achieved pre-defined safety criteria. Results suggest physical ICU rehabilitation may be safe for adults with TBI, yet the association between safety events and the timing, method and intensity of rehabilitation is difficult to determine owing to low study reporting.

In the two studies that reported the number of rehabilitation interventions and the number of adverse events, we identified an overall adverse event rate of 5.8%. Our findings support a previously published meta-analysis from general-ICU cohorts, whereby early mobilisation amongst mechanically ventilated ICU patients is deemed as safe, with no differences in adverse events or all-cause mortality compared with usual care [53, 54]. To support the delivery of safe and effect mobilisation in ICU, expert consensus papers support clinical decision making in complex ICU environments and amongst heterogenous critically ill patients [28, 29, 55]. Previously published safety data from general-ICU populations reported a safety event rate of 0.6% [27]. Although the safety rate from our analysis of patients with TBI is higher, it is important to note that the safety events reported in our meta-analysis included features that may not be attributable to rehabilitation, but rather non-specific complications of a patient’s recovery from critical illness and TBI, such as urinary tract infection, delirium or pressure ulcer. In contrast, the study of general-ICU populations focused upon specific events during rehabilitation interventions such as falls, removal of intravascular catheters and endotracheal tubes, desaturation and haemodynamic changes [27]. Adverse events and serious errors involving critically ill patients are common, occurring in approximately 20% of ICU patients, with a rate of 80.5 adverse events in 1000 patient-days [56]. Determining the safety of ICU rehabilitation specifically and accurately is therefore challenging. Nonetheless, many of the included studies in this synthesis defined specific safety and patient monitoring criteria, providing a foundation of evidence for clinical practice and the future development of safe ICU rehabilitation interventions for adults with TBI.

The results of our meta-analysis found a statistically significant increase in Perme ICU Mobility Scores in those that received ICU rehabilitation compared with those who did not. The Perme ICU Mobility Score is a tool specifically designed to measure the mobility status of ICU patients and is deemed to have good validity, inter-rater reliability and acceptability in cardiovascular ICU patients and has also been utilised in ICU studies of COVID-19 and surgical cohorts [57–60]. The score ranges from 0 to 32, judged upon 15 items across seven categories including mental status, mobility barriers, functional strength, bed mobility, transfers, gait and endurance [58]. Scores therefore require careful interpretation, as increasing scores may represent an improvement in mental status and barriers, rather than mobility itself. However, improving mobility status at the point of ICU discharge has known benefits, with increased ICU mobility independently associated with discharge to usual residence and hospital length of stay [61]. Previous studies have also confirmed the benefit of physical ICU rehabilitation upon mobility status, yet this is the first known meta-analysis specific to a TBI cohort in the ICU [33, 62]. Although statistically significant, the results of our meta-analysis are based upon two studies only, both with low participant numbers, one of which being an observational study with a high risk of bias. Nonetheless, the results show promising benefit of physical ICU rehabilitation upon mobility outcomes for adults with TBI at ICU discharge.

The results of our meta-analysis found trends to suggest that the ICU and hospital length of stay were all higher in those who received physical ICU rehabilitation compared with control groups. Although results were not statistically significant, these findings suggest potential adverse response to rehabilitation. However, the cause–effect relationship between rehabilitation and length of stay cannot be confirmed, as patients with a longer ICU admission have greater opportunity to receive rehabilitation during this period. Furthermore, owing to the nature of observational studies with unstratified experimental and control groups, the findings may be confounded by several other factors, rather than rehabilitation alone. Interestingly, those studies included in the length of stay meta-analysis also reported a statistically significant difference in the overall admission GCS scores between groups, with lower GCS (and thus more severe TBI) in the rehabilitation group compared with controls. The increased length of stay in those receiving rehabilitation may therefore be attributed to TBI severity, rather than an adverse effect of rehabilitation; however, interpretation is speculative. Differences in admission GCS and TBI severity may also explain trends in unfavourable effects upon neurological disability. However, inconsistencies in the reporting of baseline characteristics limits interpretation. Duration of admission and functional recovery may be influenced by several factors, such as severity of illness, comorbidities, associated injuries and infective complications [63, 64]. Such features were not consistently reported across the included studies, therefore their effect upon rehabilitation outcomes cannot be accurately evaluated as part of this study. Interestingly, features associated with prolonged ICU admission, such as incidence of delirium and pneumonia and duration of mechanical ventilation, have previously been shown to improve with early ICU rehabilitation [65, 66]. In contrast to our findings, previous studies in non-neurotrauma cohorts have demonstrated a benefit of rehabilitation upon duration of mechanical ventilation and ICU length of stay [65–67]. Nonetheless, owing to low study numbers, heterogeneity and non-stratified experimental and control groups, further high-quality randomised trials are necessary to confirm our findings. Future clinical trials of physical ICU rehabilitation should employ stratified intervention and control groups that account for baseline sources of confounding bias, including admission GCS, illness severity, age, sex and associated injuries, in order to enhance the validity and accuracy of subsequent research outcomes.

In addition to heterogeneous outcome measures, we also identified heterogeneity across the identified rehabilitation interventions. Our synthesis suggests that mobility interventions, using an individualised and clinically reasoned approach may be favourable for adult patients with TBI in the ICU. Previous studies in general ICU cohorts have demonstrated that mobility interventions can improve functional independence and quality of life, as well as reduce the duration of mechanical ventilation, ICU and hospital admission [31, 68]. In general medical and surgical ICU populations, mobilisation is a safe and effective ICU intervention, yet comprehensive multi-system assessment and monitoring of vital signs are essential for maintaining patient safety [27, 28, 69]. A purposeful collaboration between trauma and rehabilitation experts is therefore recommended, using a coordinated multidisciplinary approach to agitation, pain, delirium, immobility and sleep in the ICU [70].

The timing and intensity of rehabilitation are also important to consider, with previous studies investigating rehabilitation within 24 h of acute stroke, and intensive mobilisation within 72 h of admission, associating early ICU rehabilitation with adverse effects [71, 72]. The timing of initiation and intensity should therefore be based upon patient stability and safety, rather than a specific timepoint. Early mobilisation in the neurotrauma ICU has unique challenges that require careful consideration, such as haemodynamic status, cognitive impairment and impaired conscious states, as some examples [73–75]. Recent guidelines state that early mobilisation can commence within 48 h after TBI, with the caveat that mobilisation should only begin once neurosurgical, haemodynamic and respiratory stability has been achieved [70]. Furthermore, TBI recovery encompasses vast disabilities, including physical, psychological, cognitive and functional deficits, requiring extensive multi-modal and multi-disciplinary rehabilitation, beyond physical rehabilitation alone. Rehabilitation is a complex intervention and thus an individualised approach is advocated, through structured assessment and personalised rehabilitation goals [30, 76]. This systematic review provides a basis for future research, recommending an individualised approach based upon safety monitoring and comprehensive multi-component assessment.

### Limitations

This systematic review has several limitations. As grey literature was excluded, results may be subject to selection bias. However, grey literature exclusion improves the review’s methodological rigour, as all included studies have been subject to peer review. Furthermore, as only full-text studies published in the English language were included, results may be subject to publication and language bias. The external validity may also be limited, as six of the included studies are from Asia and five are from Europe, potentially limiting the application of results to international practice. Evidence selection bias may also be introduced, as only studies specific to a TBI population were included. Screening data demonstrated that 23 studies were excluded as they were not exclusively TBI, yet many other trials of general or mixed ICU cohorts could have included patients with TBI that have not been identified. Data extraction was not completed independently, but was checked by the second author, which may also introduce bias.

Whilst we recognise that rehabilitation after TBI also spans cognitive, behavioural, psychological and speech–language domains, these broader modalities fell outside the scope of our review, which focused on critically ill ICU patients where heterogeneity is already substantial. Including wider rehabilitation approaches would have increased methodological variation, reduced the interpretability of pooled analyses and been more appropriate for a scoping review than a systematic review with meta‑analysis. Given established evidence that early mobilisation and physical rehabilitation in critical care improves muscle strength, physical function and length of stay, concentrating on physical rehabilitation provided a clearer, clinically meaningful synthesis relevant to the needs of critically ill adults with TBI.

A high risk of bias across all included observational and randomised studies also limits the validity of our meta-analysis, particularly due to confounding variables, outcome measurement issues and inconsistencies in exposure to the intended intervention. Overall, results should be interpreted with caution owing to the low study numbers, low number of randomised-controlled trials and the high risk of bias, highlighting the need for further high-quality studies to confirm our findings.

## Conclusions

This is the first known systematic review investigating physical ICU rehabilitation interventions specifically for adult patients with TBI. TBI leads to devastating long-term disability, and those who present to ICU also face the deleterious consequences of critical illness upon physical, cognitive and psycho-emotional wellbeing. As such, interventions to improve patient outcomes after TBI are of significant importance. Results suggest that physical rehabilitation in the ICU is potentially safe and may improve mobility status at ICU discharge for adult patients with TBI. The low study numbers, high levels of heterogeneity and high risk of bias across the existing studies are a limiting factor to our study, highlighting that further rigorous research is urgently needed in this population. Future clinical trials require stratified intervention and control groups, randomisation with outcome assessor blinding, adequate sample size and standardised intervention development to reduce future study risk of bias. This systematic review provides a basis for future research, requiring further development of ICU mobilisation protocols utilising an individualised approach based upon safety criteria, monitoring and comprehensive multi-component assessment to inform future practice and policy for adult patients with TBI.

## Supplementary Information

Below is the link to the electronic supplementary material.

Supplementary file1 (DOCX 269 kb)

Supplementary file2 (DOCX 230 kb)

Supplementary file3 (DOCX 13 kb)

Supplementary file4 (DOCX 24 kb)
