# Supplementary material for: Rehabilitation in the Intensive Care Unit for Adults with Traumatic Brain Injury: A Systematic Review and Meta-analysis
Source: Neurocrit Care. 2026 May 23;45(2):771–86. doi: 10.1007/s12028-026-02551-x (PMC13642425; doi:10.1007/s12028-026-02551-x)
Supplement: Supplementary file 2 [file 12028_2026_2551_MOESM2_ESM.docx]

**Supplementary File 2**

**Systematic Review – Syntax & Search Terms**

Syntax

| **Syntax** | **Description** |
| --- | --- |
| **Population** | Adults, hospitalised following acute traumatic brain injury, inpatient admission to the intensive care unit (ICU) |
| **Intervention** | Any physical rehabilitation intervention delivered in the (ICU) |
| **Comparator** | No rehabilitation (can also include observational studies with no comparator) |
| **Outcome** | Any outcome measures |

Key Word Search

| **Key Word** | **Search Terms** |
| --- | --- |
| **Traumatic Brain Injury** | (‘traumatic brain injur*’ OR ‘TBI’ OR ‘brain injur*’ OR ‘head injury’ OR ‘head trauma’ OR ‘brain trauma’ OR ‘craniocerebral trauma’ OR ‘brain contusion’ OR ‘intracranial trauma’ OR ‘injur* brain’ OR ‘injur* acute brain’ OR ‘acute brain injur*’ OR ‘acute brain injur*’ OR ‘brain injur* focal’ OR ‘focal brain injur*’ OR ‘injur* focal brain’ OR ‘brain haemorrhage trauma*’ OR ‘cerebral haemorrhage trauma*’ OR ‘brain injur* diffuse’ OR ‘diffuse axonal brain injur*’ OR ‘diffuse axonal injur*’ OR ‘trauma nervous system’ OR ‘nervous system trauma’) |
| **Rehabilitation** | (‘rehab*’ OR ‘rehabilitation’ OR ‘exercise’ OR ‘exercise therapy’ OR ‘mobili*’ OR ‘mobility’ OR ‘mobilisation’ OR ‘mobilis*’ OR ‘mobiliz*’ OR ‘mobilization’ OR ‘ambulation’ OR ‘ambulate’ OR ‘physical therapy’ OR ‘physio*’ OR ‘physiotherapy’ OR ‘occupational therapy’ OR ‘PT’ OR ‘OT’ OR ‘walk* OR ‘ADL’ OR ‘activities of daily living’ OR ‘physical training’ OR ‘physical rehab*’ OR ‘exercise training’ OR ‘mobility training’ OR ‘neurological rehabilitation’ OR ‘rehab* neuro*’ OR ‘neuro* rehab*’ OR ‘rehabilitation medicine’ OR ‘physical rehabilitation medicine’ OR ‘functional rehab*’ OR ‘functional activit*’) |
| **Intensive Care Unit** | (‘intensive care unit’ OR ‘ICU’ OR ‘intensive care’ OR ‘care intensive’ OR ‘critical care unit’ OR ‘critical care’ OR ‘CCU’ OR ‘care critical’ OR ‘intensive treatment unit’ OR ‘unit intensive’ OR ‘ITU’ OR ‘high dependency unit’ OR ‘HDU’ OR ‘unit high dependency’ OR ‘intensive therapy unit’) |

Example Search Strategy
