# Supplementary material for: Rehabilitation in the Intensive Care Unit for Adults with Traumatic Brain Injury: A Systematic Review and Meta-analysis
Source: Neurocrit Care. 2026 May 23;45(2):771–86. doi: 10.1007/s12028-026-02551-x (PMC13642425; doi:10.1007/s12028-026-02551-x)
Supplement: Supplementary file 3 [file 12028_2026_2551_MOESM3_ESM.docx]

**Supplementary File 3**

**Table S3: Physical outcome measures**

Table to show the physical outcome measures reported in the included studies, categorised into themes following narrative synthesis.

| Mobility Status | Functional Status | Muscle Function | Neurological Disability | Vital Parameters | Complications Rates |
| --- | --- | --- | --- | --- | --- |
| Perme ICU Mobility Scale  Mobility Scale for Acute Stroke  Functional Ambulation Classification  Modified ICU Mobility Scale  Days to first out of bed mobilisation  ICU Mobility Scale | Barthel Index  Functional Independence Measure (FIM)  FIM Motor Score  Community Integration Questionnaire | Skeletal Muscle Index  Phase Angle | Glasgow Outcome Scale  Glasgow Outcome Scale Extended  Glasgow Coma Scale  Disability Rating Scale  Coma Recovery Scale  Early Functional Ability Score | Saturations of Oxygen  Pulse  Respiratory Rate  Blood Pressure | Pneumonia (including ventilator associated pneumonia)  Deep Vein Thrombosis  Pressure Ulcer  Pulmonary Embolism  Acute Respiratory Distress Syndrome |
