# Supplementary material for: Rehabilitation in the Intensive Care Unit for Adults with Traumatic Brain Injury: A Systematic Review and Meta-analysis
Source: Neurocrit Care. 2026 May 23;45(2):771–86. doi: 10.1007/s12028-026-02551-x (PMC13642425; doi:10.1007/s12028-026-02551-x)
Supplement: Supplementary file 4 [file 12028_2026_2551_MOESM4_ESM.docx]

**Supplementary File 4**

**Table S4: Detailed summary of rehabilitation interventions**

Intervention details including frequency, start point, dose, safety criteria, monitoring and staff for each of the included studies.

Abbreviations: ADL = activities of daily living; AFO = ankle foot orthosis; BP = blood pressure; bpm = beats per minute; CPP = cerebral perfusion pressure; cmH2O = centimetres of water; ECG = electrocardiogram; GCS = Glasgow Coma Scale; ICP = intracranial pressure; ICU = intensive care unit; MAP = mean arterial pressure; mmHg = millimetres of mercury; PEEP = positive end expiratory pressure; ROM = range of movement; RR = respiratory rate; SpO2 = saturations of oxygen

| **First Author**  Surname, Year (reference) | **Intervention Details** | **Frequency** | **Start Point** | **Dose** | **Safety criteria and monitoring** | **Staff** |
| --- | --- | --- | --- | --- | --- | --- |
| **Alvsåker, 2023** | Early interdisciplinary rehabilitation including three elements of training: (1) ADLs, (2) Mobility, (3) Stimulation of swallowing and eating/ drinking | Twice daily. | After 72 hours, once short-acting opioid infusions were discontinued, after ICU team assessment. | 45-60 minutes per session. | ICU nurses monitored patient vital parameters. | Physiotherapist;  Occupational Therapist |
| **Alvsåker, 2025** | Three rehabilitation components added to our standard physiotherapy: (1) ADL training, (2) Mobility training, (3) Stimulation of swallowing and eating/ drinking. Rehabilitation strategies were tailored to suit each patient based upon their current health condition. | Twice daily. | After 72 hours, once short‐acting opioid infusions were discontinued and either extubated or tracheostomized. | 30-60 minutes per session. | ICU nurses monitored patient vital parameters. | Rehabilitation Physician; Physiotherapist;  Occupational Therapist |
| **Cetin, 2024** | Patients were assigned individual rehabilitation programs, aiming to prevent complications, minimize impairments in body structure and function, and increase independence in ADLs. The program varied depending on the patient’s consciousness. For GCS 9-15: (1) Active / active assisted ROM, (2) Functional extremity patterns, (3) Mobility based interventions, (4) Balance training. For GCS 3-8: (1) Passive ROM, (2) Joint mobilisation, stretching and massage, (3) Antispastic positioning and functional patterns | Five days weekly. | Time from admission to rehabilitation consultation = 19.82 +/- 17.9 days. | 60 minutes per session. | Patient’s normal vital signs were monitored by a neurosurgeon. Exclusion = spinal trauma including vertebral fracture affecting the spinal cord. | Physiotherapist |
| **Elkbuli, 2022** | Physical therapy / mobilisation (nil further details recorded). | NR | Four groups based on timing: either <24 hours, 24-48 hours, 48-72 hours, or >72 hours. | NR | NR | Physical Therapists |
| **Lima, 2022** | Level 1: passive limb exercises for sedated patients. Level 2: exercises in a seated position in patients without sedation, or 24 hours after sedation suspension. This level was subdivided, according to consciousness, collaboration and understanding. Level 2A was applied in non-sedated patients without collaboration and/or understanding; (1) Passive exercises, (2) Cervical and trunk exercises, (3) Weight discharge in upper limb. Level 2B was applied in collaborative patients who understood commands; (1) Pro-active exercises, (2) Cervical and trunk exercises, (3) Weight discharge in upper limb, (4) Progress to standing and gait training. | Once a day, 5-days a week (excluding weekends). | 24 hours after ICU admission.  The presence or absence of sedatives, vasoactive drugs, hemodynamic aspects and clinical conditions were assessed to determine initiation. | 30 minutes per session. | Mobilisation was interrupted based on: (1) Systolic BP <90 mmHg; (2) ICP > 22 mmHg; (3) Heart rate <50 bpm or >140 bpm; (4) Vasoactive drugs > 5 mg/min; (5) PEEP > 12 cm H2O; (6) Peripheral oxygen saturation < 90%; (7) Respiratory rate > 40 breaths per minute; (8) Axillary temperature > 39 ºC. Patients with orthopaedic external fixators, musculoskeletal traction, lower limb amputation, open abdominal wound, spinal cord lesions were excluded. | Physicians;  Physical Therapists |
| **Lippert-Grüner, 2010** | Interventions were scheduled individually, including the following; (1) Facial-oral tract therapy to re-establish swallowing, (2) Physiotherapy (physio) for sensorimotor functions, (3) Speech therapy for simple level of communication, (4) Occupational therapy (OT) for independence in ADLs, (5) Neuropsychology treatment of cognitive and behavioural deficits. | Daily | After controlled mechanical ventilation was completed and circulatory functions and ICP had normalized. | Patient specific, on average 4-5 hours daily. | Initiated once mechanical ventilation and sedation was completed and circulatory functions had normalized including cardiopulmonary and ICP stability with no severe infections. | Physiotherapy; Speech Therapy;  Occupational Therapy. |
| **Mehta, 2017** | Positioning regimes in bed, including: (1) Supine lying, (2) Left lateral lying, (3) Right lateral, (4) Reclined sitting (30°–70°). Positions were modified as needed for patients with craniotomy and other injuries (such as fracture clavicle)). | One session only in one day. | Once haemodynamically stable | 15 minutes | Patient monitoring included: ECG rhythm, SpO2, BP and respiratory rate. If patient felt discomfort or was hemodynamically unstable, then treatment would stop. Exclusion = fractures of the pelvis, spine, shoulder girdle, ribs, or long bones, spinal cord injury, hemodynamically unstable (hypertensive, acute myocardial infarction, unstable angina), any past history of cardiac or thoracic surgery. | Physiotherapist |
| **Nagarathinam, 2019** | Mobilization to sitting i.e. bringing the patient to an upright sitting position from supine lying passively on to a chair. After receiving routine physiotherapy, subjects were mobilized passively to sitting in a chair, positioned with pillows and straps in comfortable position. | 7 days a week for 15 days. | Haemodynamically stable off sedation. | 30 minutes vs  1-hour sitting. | BP and heart rate monitored. Patients were transferred back to bed if they showed syncope or pre-syncopal symptoms. Exclusion = severe cardiac arrhythmia, uncontrolled seizure and pelvis fracture. | Physiotherapist |
| **Ribberholt, 2021** | Early orthostatic exercise consisted of daily exercise on an ERIGO basic® tilt-table (Hocoma AG, Switzerland) to 70° head-up tilt for 20 minutes administered by two physiotherapists and a nurse. The ERIGO basic® has a built-in robotic stepping device intended to counteract drop in blood pressure (set at 50–60 steps per minute). | Daily, 5-days a week (Monday to Friday). | With stable ICP <20mmHg for 24 hours prior to inclusion. | 20 minutes | Reduction in BP, cerebral perfusion pressure or increase in heart rate and ICP beyond predetermined limits. Exclusion = spinal cord injury or fractures of the lower extremities that prohibited weight-bearing. | Physiotherapist;  Nurse |
| **Steiner, 2016** | Basal stimulation including occupational therapy, physiotherapy, facio-oral therapy, and speech therapy, (2) Prevention of secondary complications like muscle contractions and infections, (3) Goals and rehabilitation regime established, (4) Psychosocial mentoring of the family members to provide positive input | NR | After the acute phase of trauma and once haemodynamically stable (even under catecholamines) with stable ICP and CPP. | NR | Complete paraplegic patients or those with more than two concomitant injuries with impact on the progress of the patient were excluded. | Physiotherapist;  Speech Therapy;  Occupational Therapy |
| **Wu, 2024** | Level 1: the head of the bed is elevated to 30–60;  Level 2: the head of the bed is elevated to 90  Level 3: sitting on bed; Level 4: sitting on the side of the bed with help; Level 5: sitting on the side of the bed no help; Level 6: sitting on a wheelchair;  Level 7: standing with help; Level 8: standing without help; Level 9: walking with help;  Level 10: walking without help. The level was based on state of consciousness: GCS ≤8 = up to level 3; GCS 8-12 = up to level 7, GCS >12 = all levels of intensity. | NR | Assessed within 24 hours. | 2-4 hours per day. | Patient vital signs, consciousness, and pupil change were monitored. Start and safety criteria included the following: Heart rate <60 or >120bpm; MAP <70 or >110 mmHg;  Respiratory rate <5 or >40; SpO2 <90%; ICP >20mmHg. Exclusion = severe trauma to other organs, spinal fracture, serious fracture and unable to change position, acute myocardial infarction, arrythmia, arterial dissection, serious cardiovascular disease, deep vein thrombosis, or uncontrolled epilepsy. | Rehabilitator;  Nurse |
| **Yen, 2022** | Intervention followed the Modified ICU Mobility Scale: Level 0: lying in bed, no activity; Level 1: head of the bed elevated to 45–60 with passive/active assisted exercises; Level 2: head of the bed elevated to 90 with active assisted and head/trunk control training; Level 3: sitting on the edge of the bed with sitting balance training; Level 4: standing with balance training; Level 5: transfer from bed to wheelchair; Level 6: marching in place at bedside; Level 7: walking with assistance of 2 or more; Level 8: walking with assistance of 1 person;  Level 9: walking independently with an aid; Level 10: walking independently without an aid. | Five times per week | Within 7 days after injury, once deemed hemodynamically, medically and neurosurgically stable by the trauma ICU team physician. | 20-30 minutes per session | The safety criteria included:  1) Cardiovascular: heart rate 40-130bpm, systolic BP 90-180, MAP 60-110.  2) Respiratory: RR 5-40, Sa02 >99%, if on a ventilator with Fi02 <60% and PEEP <10. 3) Neuro: GCS score ≥6 and ICP <20. Any serious adverse events led to immediate suspension (including falls, unplanned extubation, cardiac arrest, tube dislodgement, and atrial or ventricular tachycardia/ arrhythmia). Exclusion = unstable fractures or injury requiring bed rest, open wounds, active organ bleeding, open chest/ abdomen, transfusion protocol, active myocardial infarction, uncontrolled seizures, aortic dissection. | Physical Therapists |
| **Yen, 2024** | Intervention followed the Modified ICU Mobility Scale: Level 0: lying in bed, no activity; Level 1: head of the bed elevated to 45–60 with passive/active assisted exercises; Level 2: head of the bed elevated to 90 with active assisted and head/trunk control training; Level 3: sitting on the edge of the bed with sitting balance training; Level 4: standing with balance training; Level 5: transfer from bed to wheelchair; Level 6: marching in place at bedside; Level 7: walking with assistance of 2 or more; Level 8: walking with assistance of 1 person;  Level 9: walking independently with an aid; Level 10: walking independently without an aid. Exercise intensity was tailored based on physiological response, with adjustments made by the physiotherapist. | Daily | Within 7 days after injury. | 30 minutes | Vital signs were monitored during sessions by the primary nurse who ensured secure tube connections and performed essential interventions such as sputum suctioning. Safety criteria included: 1) Respiratory: Sp02 > 92%, Fi02 ≤ 60%, PEEP ≤ 10 cmH2O; 2) Cardiovascular: resting heart rate ≤ 130 bpm and not using high-dose vasopressor (> 0.2μg kg−1 min−1). Exclusion: persistent ICP >22mmHg, uncontrolled seizures, significant bleeding, ruptured or leaking aortic aneurysm, acute myocardial infarction or order that required immobilisation. | Physiotherapist |
